# Comparative Evaluation of Different Co-Antioxidants on the Photochemical- and Functional-Stability of Epigallocatechin-3-gallate in Topical Creams Exposed to Simulated Sunlight

**DOI:** 10.3390/molecules18010574

**Published:** 2013-01-04

**Authors:** Santo Scalia, Nicola Marchetti, Anna Bianchi

**Affiliations:** Department of Chemical and Pharmaceutical Sciences, University of Ferrara, via Fossato di Mortara 17, 44121, Ferrara, Italy

**Keywords:** epigallocatechin-3-gallate, photodegradation, photostabilizers, topical emulsions, *in vitro* antioxidant activity, co-antioxidants

## Abstract

The catechin (−)-epigallocatechin-3-gallate (EGCG) exhibits high antioxidant activity and it has been reported to provide protection of the skin against damage induced by solar UV radiation. However, EGCG is highly unstable under sunlight. The present study aimed to compare the effectiveness of the co-antioxidant agents vitamin E, butylated hydroxytoluene, vitamin C and α-lipoic acid for their potential to protect the catechin from photochemical degradation. Model creams (oil-in-water emulsions) containing EGCG (1%, w/w) alone or combined with equimolar concentrations of co-antioxidant were exposed to a solar simulator at an irradiance corresponding to natural sunlight. Photodegradation was evaluated by HPLC-UV and HPLC-ESI-MS/MS. Addition of the co-antioxidants vitamin C and α-lipoic acid to the formulation significantly reduced the light-induced decomposition of EGCG from 76.9 ± 4.6% to 20.4 ± 2.7% and 12.6 ± 1.6%, respectively. Conversely, butylated hydroxytoluene had no effect (EGCG loss, 78.1 ± 4.6%) and vitamin E enhanced the EGCG photolysis to 84.5 ± 3.4%. The functional stability of the catechin in the creams exposed to the solar simulator was also evaluated by measuring the *in vitro* antioxidant activity. Following irradiation, the reduction of the EGCG formulation antioxidant power was lower (21.8%) than the extent of degradation (76.9%), suggesting the formation of photoproducts with antioxidant properties. The influence of the examined co-antioxidants on the functional stability of the catechin under simulated sunlight paralleled that measured for the EGCG photodecomposition, α-lipoic acid exerting the greatest stabilising effect (antioxidant activity decrease, 1.4%). These results demonstrated that α-lipoic acid is an effective co-antioxidant agent for the stabilization of EGCG in dermatological products for skin photoprotection.

## 1. Introduction

(−)-Epigallocatechin-3-gallate (EGCG) is the most abundant and biologically active catechin in green tea [1,2]. Numerous studies have described its beneficial pharmacological effects, including potent antioxidant activity, prevention against several diseases (e.g., cardiovascular and neuro-degenerative pathologies and certain cancers) and protection of the skin from the damage caused by the increased formation of free radicals induced by exposure to solar UV radiation [2,3,4,5,6,7].

More specifically, topical application of EGCG has been shown to inhibit UV-B (290–320 nm)- induced cutaneous tumors in mice [8] and to reduce in animal and human skin the inflammatory processes (e.g., increase production of prostaglandin metabolites, infiltration of inflammatory leukocytes and erythema) triggered by exposure to the sun [9]. Moreover, topical treatment with EGCG has been reported to protect the cutaneous immune system from damage caused by sunlight and to prevent the expression of matrix metallo-proteinases induced by solar UV radiation [5,10].

However, the therapeutic applicability of this cathechin is limited by its high reactivity, leading to oxidation, hydrolysis, epimerization and polymerization reactions [11,12,13], which represent a major challenge for the formulation of EGCG into skin care products. Many reports in the literature have examined the chemical instability of EGCG in dermatological preparations [12,13,14,15,16] and several strategies have been proposed to reduce the catechin decomposition, including the use of acidic media [15], micro- and nano-encapsulation [13,17] and, especially, the addition of co-antioxidants [13,14,15,16].

The effect of light on EGCG has not been studied in as much detail as for the catechin chemical decomposition. This is a disadvantage, since an essential requirement for the potential use of EGCG as topical skin photoprotectant is an adequate stability under solar irradiation. A recent investigation by this group demonstrated, for the first time, that EGCG undergoes rapid and marked degradation (>68% loss) in model dermatological formulations (emulsions) exposed to UV radiation [18]. In addition, the study reported on the relative ability of sunscreen agents to prevent the catechin photolysis, which was reduced by ca. 50% in the presence of a molar excess (3 fold) of the water soluble UV-B filter, benzophenone-4 [18].

In order to gain further information on the catechin photochemical behaviour, in the present investigation we have systematically examined the influence of some the most commonly used antioxidant agents (vitamin E, butylated hydroxytoluene, vitamin C and α-lipoic acid) on the light-induced decomposition of EGCG in model topical preparations (oil-in-water emulsions). The photostability of each examined co-antioxidants was also measured. Moreover, the *in vitro* evaluation of the functional stability (*i.e.*, antioxidant activity) of the studied formulations, before and after irradiation, is reported.

## 2. Results and Discussion

Since the incorporation of antioxidants in the formulations containing EGCG is the most common approach to protect the cathechin from chemical degradation [13,14,15,16], it is interesting to investigate whether they exerted any effect also on the stability of EGCG under sunlight exposure. Vitamin E, BHT and vitamin C were selected as reducing agents, since they have been reported to provide high levels of protection to EGCG in long-term stability studies [14,15,16,19]. In addition, the effect of α-lipoic acid, a potent radical scavenger [20,21] was also examined.

### 2.1. Photodegradation Studies

For the photodegradation studies, a hydrophilic cream (oil-in-water emulsion) was used as a vehicle, since it represents the most commonly employed topical preparation [22]. The pH of the cream was adjusted to 5, because this is the normal value for dermatological products [22] and at this pH, EGCG exhibits sufficient chemical stability [15].

**Figure 1:**
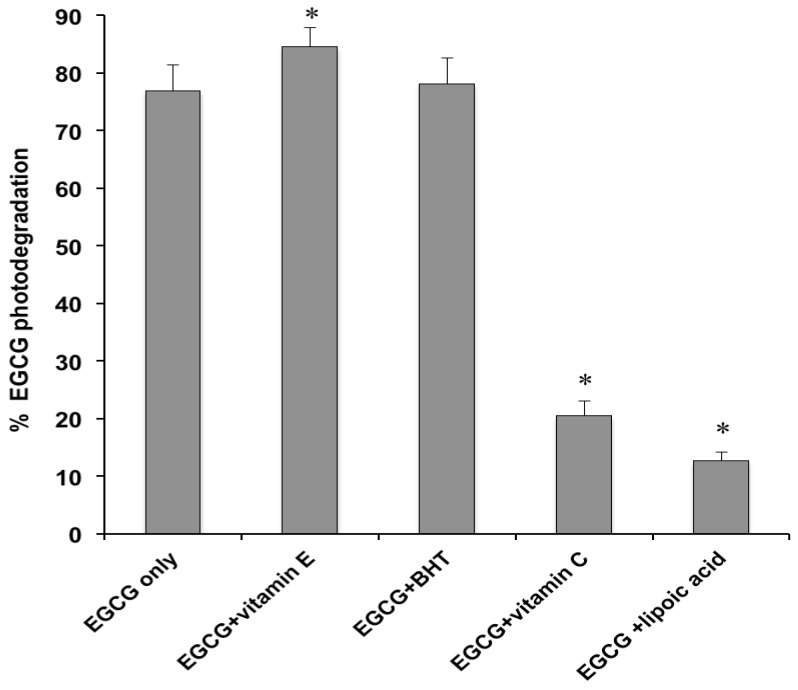
EGCG photodegradation (%) in its formulations, without or with co-antioxidants (EGCG/co-antioxidant molar ratio, 1), after 1 h irradiation with the solar simulator. Each value is the mean ± S.D. of at least six experiments. * *p* < 0.05 (ANOVA) *vs*. EGCG only.

The emulsions containing EGCG (1.0%) in the absence or in the presence of equimolar concentrations of the examined co-antioxidants were exposed to the solar simulator at an irradiance comparable to natural sunlight and the extent of catechin degradation was measured by HPLC. As illustrated in Figure 1, 76.9 ± 4.6% of EGCG was lost following irradiation of the cream containing no co-antioxidants. This result is in good agreement with a previously reported study [18]. Additional photodegradation experiments were performed at lower solar simulator emission (350 W/m^2^). Under these conditions, the EGCG photodecomposition was reduced to 62.2 ± 3.7%. However, subsequent experimentation was performed at 500 W/m^2^, since this value corresponds to an UV irradiance similar to real sun exposure [18]. Supplementation of the EGCG preparation with equimolar concentrations of the reducing agents vitamin E or BHT, did not decrease the light-induced degradation of EGCG, which was actually enhanced to 84.5 ± 3.4% and 78.1 ± 4.6% in the presence of vitamin E and BHT (Figure 1), respectively. This is in line with earlier preliminary findings [18]. However, the observed differences were significant for vitamin E only (ANOVA and Tukey’s post test, *p* < 0.05). On the contrary, a marked reduction of the photodecomposition of the catechin to 20.4 ± 2.7% and 12.6 ± 1.6% was observed for the creams incorporating equimolar concentrations of the co-antioxidants vitamin C and α-lipoic acid, respectively (Figure 1).

**Figure 2:**
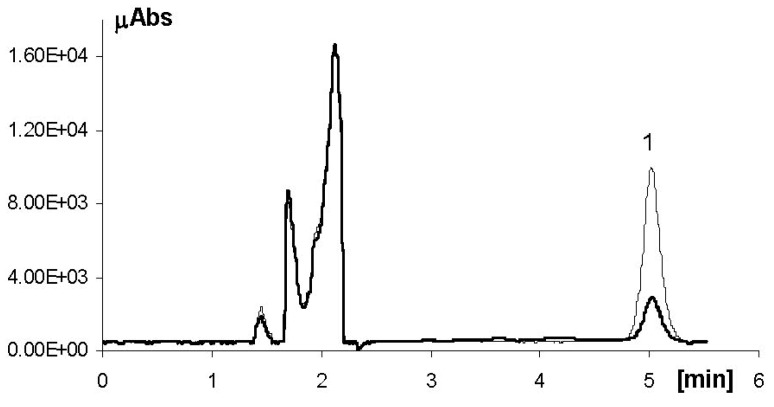
Representative HPLC-UV chromatogram of a cream preparation containing EGCG before (thin line) and after (thick line) 1 h irradiation with the solar simulator. Peak 1 = EGCG.

**Figure 3:**
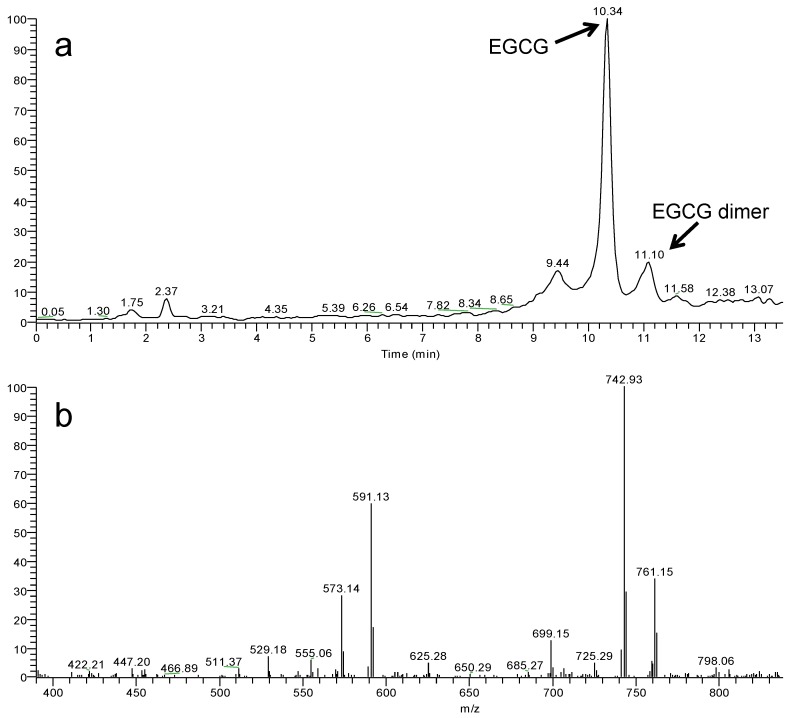
(a) Extracted ion current trace obtained by HPLC-ESI-MS/MS analysis of a cream preparation irradiated with the solar simulator. (b) ESI negative HPLC-MS/MS spectrum of EGCG dimer peak: characteristic daughter ions from CID fragmentation of [M−H]^−^ at *m/z* 913 are 573.1, 591.1, 742.9 and 761.1 *m/z*.

No peaks traceable to photodegradation products were observed in the HPLC-UV chromatogram of the irradiated sample (Figure 2). HPLC-ESI-MS/MS analysis of the same sample with gradient elution (Figure 3a), revealed the presence of a peak, which on the basis of the molecular ion mass (*m/z* 913) and the major mass fragments (*m/z* 573, 591, 743, 761) (Figure 3b), was tentatively identified as a dimer of EGCG [23]. Although further studies are required to confirm this result, this represents the first report on the identification of EGCG photoproducts.

Photolysis experiments, carried out after 4-month storage of the formulations at room temperature and in the dark, showed values for EGCG photodecomposition (data not shown) superimposable to those reported in Figure 1, thus indicating that the effect of the studied reducing agents was maintained after the above time period. 

Although several studies have demonstrated that the addition of co-antioxidants (*i.e.*, substances which are preferentially oxidized in place of the catechin) to EGCG formulations is an effective approach for protecting the catechin against chemical degradation [14,15,16,19], the data obtained in the present investigation indicated that these additives can produce conflicting and distinctly different effects on the stability of EGCG under light exposure. More specifically, BHT was ineffective and vitamin E actually increased the catechin photolability, instead of stabilizing it. This phenomenon could be traced to generation of radical species by these reducing agents upon UV absorption (e.g., tocopheryl radical) [24,25], that depleted EGCG. On the other hand, efficient photostabilization of EGCG was achieved by vitamin C and α-lipoic acid, leading to a 74–84% reduction in the light-induced decomposition of the catechin. Although, irradiation of vitamin C can also produce free radicals such as the ascorbyl radical, its low reactivity and redox potential (0.28 V) [13,26] enable protection of EGCG (redox potential, 0.43 V) from oxidation. The significantly (*p* < 0.05) higher stabilizing activity of α-lipoic acid, compared to vitamin C (Figure 1), could be ascribed to hydrogen transfer (α-lipoic acid is converted to dihydrolipoic acid upon UV irradiation) [27] to the catechin semiquinone intermediate, reversing it back to the reduced state, as it has been reported for other thiol reducing agents [13].

Moreover, the obtained data indicated that photostabilization of EGCG is unrelated to competitive UV absorption by the co-antioxidants. In fact, despite the more efficient spectral overlapping (absorption in the same wavelength region as EGCG, λ_max_, ca.280 nm) by vitamin E (λ_max_, ca.290 nm) and BHT (λ_max_, ca. 285 nm) as compared to vitamin C (λ_max_, ca.260 nm) and α-lipoic acid (λ_max_, ca.330 nm), only the latter co-antioxidants produced a stabilizing effect on the illuminated catechin.

**Figure 4:**
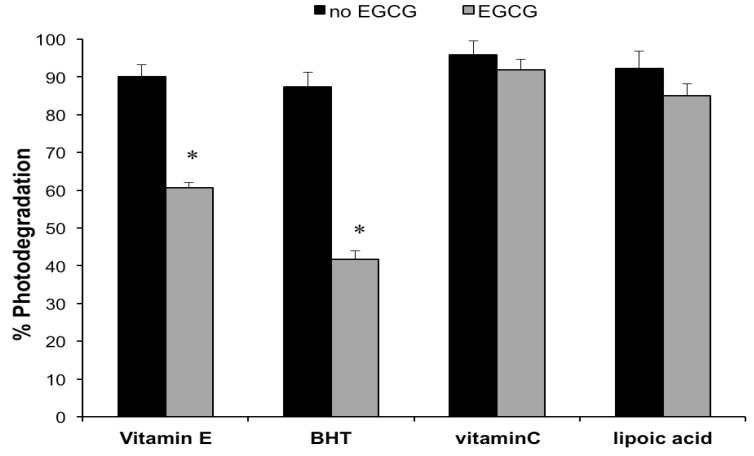
Photodegradation (%) of the co-antioxidants, vitamin E, BHT, vitamin C or α-lipoic acid in their formulations withouth or with EGCG (1%), after 1 h irradiation with the solar simulator. Values are means ± S.D. of at least six experiments. * *p* < 0.05 (*t*-test).

In order to acquire further information on the contrasting effects exerted by the examined co-antioxidants (Figure 1), the same formulations submitted to the photolysis studies were also assayed for their co-antioxidant concentrations, before and after exposure to the solar simulator. In addition, creams containing each co-antioxidant, without the catechin, were also subjected to irradiation. The percentage losses measured for the reducing agents, in the presence and in absence of EGCG, are illustrated in Figure 4. When the catechin was not incorporated into the emulsions, the degree of photodegradation was high (>87.4%) and similar for all studied co-antioxidants. In the presence of EGCG, the light-induced decomposition of vitamin E and BHT was decreased by 32.8–52.3%, whereas no significant differences were measured for vitamin C and α-lipoic acid. These data support the hypothesis that the enhanced EGCG photolability observed in the creams containing vitamin E and BHT (Figure 1), is due to their regeneration by the catechin.

### 2.2. Antioxidant Activity

In order to assess whether the photodecomposition of EGCG results in an equivalent loss of potency, the functional stability of the catechin in the formulations exposed to simulated sunlight was evaluated by measuring the *in vitro* antioxidant properties. The widely used 1,1-diphenyl-2-picrylhydrazyl (DPPH) assay [28] was selected for the determination of the antioxidant activity. Such methodology has been previously employed to assess the antioxidant capacity of semisolid formulations [29,30]. The DPPH radical is a stable free radical with strong absorption at 517 nm, which can be scavenged by electron or hydrogen donation from the antioxidant, leading to a decrease of its absorbance.

**Figure 5:**
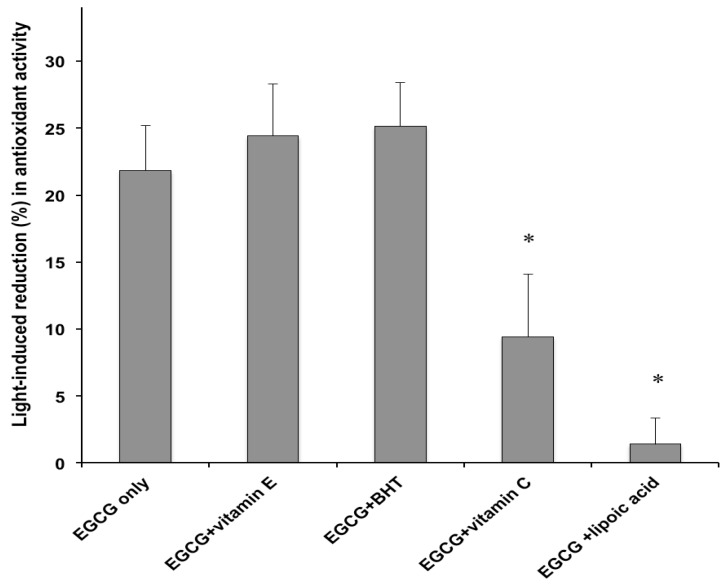
Antioxidant activity decrease (%) of formulations containing EGCG without or with co-antioxidants (EGCG/co-antioxidant molar ratio, 1), after 1 h irradiation with the solar simulator. Each value is the mean ± S.D. of at least six experiments. * *p* < 0.05 (ANOVA) *vs*. EGCG only.

**Figure 6:**
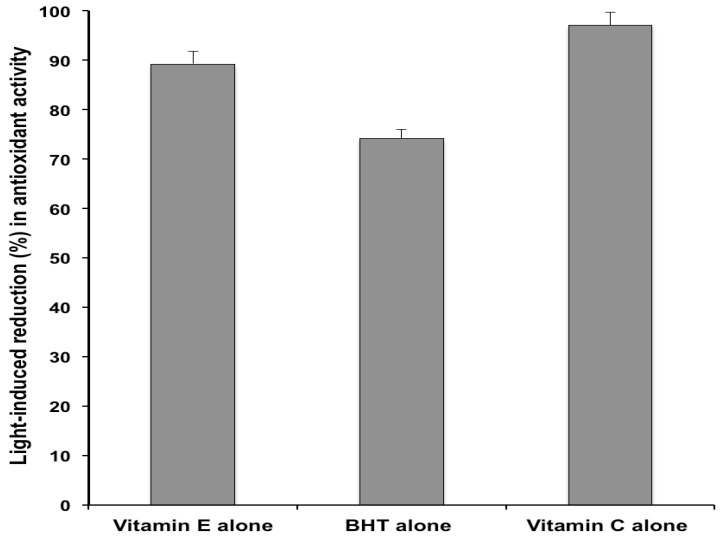
Antioxidant activity reduction (%) of formulations containing vitamin E, BHT or vitamin C as the only antioxidant, after 1 h irradiation with the solar simulator. Each value is the mean ± S.D. of at least six experiments.

The same formulations submitted to the photodegradation experiments were subjected to the DPPH antioxidant assay. Preliminary studies were performed by comparing the DPPH radical scavenging activity of the tested cream and a methanolic solution containing an equivalent catechin concentration. No significant differences were observed, thus ruling out interferences from the formulation excipients. Figure 5 displays the percentage variation in DPPH radical scavenging capacities of the studied emulsions following exposure to the solar simulator. Irradiation of the cream containing EGCG only, caused a 21.8 ± 3.4% reduction of the antioxidant activity. Since photolysis of the same formulation led to a 76.9 % decrease of the initial catechin concentration (Figure 1), it is suggested that the light-induced EGCG degradation generated products that possess antioxidant properties. For instance, it has been shown that catechin dimers originating from chemical oxidation of monomeric catechins exhibit antioxidant activity, although they are less potent than the precursor compounds [31]. Moreover, the results illustrated in Figure 5, indicated that the effect of the examined co-antioxidants on the DPPH radical scavenging capacity of the irradiated EGCG formulations parallels that measured for the catechin photodecomposition (Figure 1). In particular, vitamin E and BHT enhanced the light-induced loss of the EGCG formulation antioxidant power (from 21.8% to 24.4% and 25.1%), although the differences were not statistically significant (Figure 5). On the other hand, the functional stability of the catechin under simulated sunlight was significantly improved by addition of vitamin C and α-lipoic acid, which achieved a marked reduction in EGCG photochemical decomposition (Figure 1). After irradiation, the percentage loss of EGCG antioxidant activity was reduced from 21.8% (control formulation without co-antioxidants) to 6.3% and 1.4% in the creams containing vitamin C and α-lipoic acid, respectively (Figure 5). The observed differences were statistically significant. In accordance with the data on the light-induced EGCG decomposition (Figure 1), α-lipoic acid exerted the greatest stabilizing effect on the antioxidant activity of the catechin upon exposure to solar UV radiation. Also for the emulsions containing EGCG combined with the co-antioxidants, the reduction in antioxidant power (Figure 5) was markedly lower than the extent of photodegradation (Figure 1). In order to verify whether the discrepancy between the extent of photodegradation and the loss of functional stability applied also to the formulations without EGCG, the DPPH radical scavenging capacity of the creams containing vitamin E, BHT or vitamin C, as the only antioxidants, was measured. Under the experimental conditions of the *in vitro* antioxidant assay, it was not possible to evaluate the antioxidant activity of the cream containing α-lipoic acid alone, due to unfavourable interaction between α-lipoic acid and DPPH radical [32]. The data, reported in Figure 6, show for the creams without the catechin a good agreement between the decrease in antioxidant power and the percentage photodecomposition values (Figure 4).

This indicated a distinct difference between the behavior under sunlight of EGCG and the other examined antioxidants. Namely, for the latter, the light-induced reduction in the initial concentration led to an equivalent decrease in activity, whereas for EGCG the loss of antioxidant capacity was much lower than the extent of photodecomposition.

The results illustrated in Figure 4 and Figure 6 pointed out, in line with former findings [24,33], the extreme photolability of the individual co-antioxidant (vitamin E, BHT, vitamin C and α-lipoic acid) in their formulations. As these reducing agents are commonly used in many sun-care products [33,34,35], their real effectiveness during exposure to solar radiation is questionable, since their cutaneous photoprotective activity (deactivation of radical generated by sunlight) will be rapidly lost, due to their marked photodegradation. Accordingly, in order to elicit their effect, application of antioxidant combinations and treatment prior or after UV exposure have been suggested [24,33,34,35].

## 3. Experimental

### 3.1. Materials

EGCG (purity > 95%) was from DSM (Basel, Switzerland). Butylated hydroxytoluene (BHT), vitamin E, vitamin C and α-lipoic acid as well as the excipients for the cream preparations were supplied by Seppic (Paris, France) and ACEF (Piacenza, Italy). Methanol, acetonitrile and water were high-performance liquid chromatography (HPLC)-grade from Merck (Darmstadt, Germany). 1,1-Diphenyl-2-picrylhydrazyl (DPPH) was purchased from Sigma Aldrich (Steinheim, Germany). All other reagents and solvents were of analytical grade (Sigma).

### 3.2. High-Performance Liquid Chromatography

The HPLC apparatus consisted of a modular chromatographic system (Model 1580-PU pump and Model 975-UV variable wavelength UV-Vis detector; Jasco, Tokyo, Japan) linked to an injection valve with a 20 µL sample loop (Model 7725i Rheodyne, Cotati, CA, USA). Data acquisition and processing were accomplished with a personal computer using Borwin software (JBMS Developpements, Le Fontanil, France). Sample injections were effected with a Model 701 syringe (10 µL; Hamilton, Bonaduz, Switzerland). Chromatography was performed on a 5-µm Luna C18 column (150 × 4.6 mm i.d.; Phenomenex, Torrance, CA, USA) fitted with a guard column (5-μm particles, 4 × 3 mm i.d.) and eluted isocratically at a flow-rate of 1.0 mL/min. For EGCG, the mobile phase was sodium phosphate buffer (pH 2.8; 0.03 M)—acetonitrile (82:18, v/v) and the detector was set at 280 nm, in accordance with a recently developed method [18]. Vitamin E analysis was carried out, as previously described [36], with a methanol/acetonitrile (75:25, v/v) eluent monitored at 290 nm. For BHT separation, methanol/water (92:8, v/v) was used as the mobile phase with UV detection at 280 nm [37]. The chromatographic conditions for α-lipoic acid comprised sodium phosphate buffer (pH 2.8; 0.03 M)/acetonitrile (60:40, v/v) as the eluent and detection at 336 nm [38]. Vitamin C was analyzed according to Heudi *et al.* [39] with an aqueous solution of trifluoroacetic acid (0.025%; pH 2.6) as the mobile phase and UV detection at 254 nm. The identity of EGCG, vitamin E, vitamin C, BHT and α-lipoic acid peaks were assigned by co-chromatography with the authentic standards. Quantification was carried out by integration of the peak areas using the external standardization method.

### 3.3. HPLC-Tandem Mass Spectrometry

The HPLC- tandem mass spectrometric (HPLC-MS/MS) system employed was a modular Surveyor liquid chromatograph (Thermo Scientific, Waltham, MA, USA) equipped with an autosampler, a quaternary micro pump and a 2.5-μm Luna C18 HST column (100 × 2.1 mm; Phenomenex) eluted, at a flow rate of 0.1 mL/min, with a linear gradient from 10 to 90% of methanol in formate buffer. The column was coupled with a LTQ XL (Thermo Scientific) linear ion trap mass spectrometer. The electrospray ionization (ESI) interface was operated under both positive (ESI+) and negative (ESI^−^) ionization mode, with applied spray voltages of +4.8 kV and −4.8 kV, respectively. Capillary temperature was 270 °C, tube lens 75 V (ESI+) or −75 V (ESI^−^), ion transfer capillary 5 V (ESI+) or −5 V (ESI^−^). The intensities for both EGCG and dimer ions were higher in negative ESI mode.

### 3.4. Emulsion Formulations

Photostability studies were performed on cream preparations (oil-in-water emulsions) containing 1% (w/w) EGCG. The emulsion excipients were: cetearyl alcohol (1.5%), glyceryl monostearate (1.5%), sweet almond oil (5.0%), cetearyl isononanoate (5.0%), dimethicone (0.5%), Phenonip (0.8%; phenoxyethanol and parabens), Montanov 82 (5.0%; emulsifying agent based on cetearyl alcohol and cocoglucoside) for the internal oil phase and propylene glycol (5.0%), EDTA (0.1%), sodium dehydroacetate (0.1%), citric acid (qs pH 5) and deionized water (qs 100%) for the external phase. The selection of the excipients was based on a previous study on the formulation of emulsions containing EGCG [18]. The creams were prepared according to the common procedure used in compounding practice. EGCG (solubilized in propylene glycol) was added in the cooling phase of the emulsion formulation at about 35 °C. Creams containing the catechin in conjunction with equimolar concentrations of vitamin E (solubilized in sweet almond oil and added to the finished emulsion at room temperature), BHT (dissolved in the oil phase), vitamin C (dissolved in deionized water and added to the finished emulsion) and α-lipoic acid (dissolved in sweet almond oil and added to the finished emulsion) were also prepared. Moreover, creams containing equivalent amounts of vitamin E, BHT, vitamin C or α-lipoic acid, as the only antioxidant agent (*i.e.*, without EGCG), were also formulated and examined.

### 3.5. Photodegradation Studies

Portions (ca. 40 mg) of the cream preparations were evenly spread by means of a syringe onto the bottom of beakers (surface area, 16.0 cm^2^). The samples were secured by gumming them to a support and then irradiated for 1 h with a solar simulator (Suntest CPS+, Atlas, Linsengericht, Germany) equipped with a Xenon lamp, an optical filter to cut off wavelengths shorter than 290 nm, an IR-block filter to avoid thermal effects and an air cooling system. The solar simulator emission was maintained at 500 W/m^2^, corresponding to an UV irradiance of 54.9 W/m^2^ (irradiation dose, 198 kJ/m^2^), comparable with natural sunlight whose irradiance ranges between ca. 10 W/m^2^ (cloudy) and ca. 60 W/m^2^ (sunny day) [40]. The temperature inside the solar simulator during irradiation never exceeded 38 °C. After the exposure interval, the samples were quantitatively transferred into a 20-mL calibrated flask with methanol (2 × 8 mL), subjected to sonication (10 min) and analysed by HPLC after dilution to volume (20 mL) and filtration (0.45 μm membrane filters). For the assay of vitamin C, extraction was performed under the same conditions reported above using water instead of methanol. The degree of photodegradation was evaluated by measuring the percentage of recovered EGCG with respect to non-irradiated samples. The results were the average of at least six experiments.

### 3.6. Antioxidant Activity

The *in vitro* evaluation of the antioxidant activity was performed on a portion of the same methanolic solution used for the photodegradation assay and obtained by extraction of the examined creams, before and after exposure to the solar simulator (see Section 3.5). The antioxidant activity was measured by the DPPH assay, according to the method of Fukumoto and Mazza [41], with minor modifications. Aliquots (0.5 mL) of the test samples from the studied formulations, were added to 1.5 mL of the DPPH stock solution (0.1 mM in methanol). The mixture was stirred vigorously and incubated for 30 min in the dark at room temperature. Then, the sample absorbance was measured at 517 nm (Uvikon 923 spectrophotometer, Kontron Instrument, Zurich, Switzerland). The control solution contained the same concentration of DPPH in methanol. The DPPH radical scavenging activity of the antioxidant formulations was calculated according to the following equation:

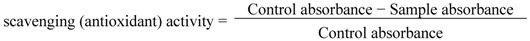


Samples were tested in sextuplicate.

### 3.7. Statistical Analysis

Analysis of data was performed using Student’s *t*-test, analysis of variance (ANOVA) and Tukey’s post-test. *P*-values < 0.05 were considered to be significant. All computations were carried out using the statistical software GraphPad Instat (Graphpad Software, San Diego, CA, USA).

## 4. Conclusions

The use of co-antioxidants represents the most common approach to overcome the problem of the chemical instability of EGCG in its formulations. However, in order to ensure the efficacy of EGCG dermatological preparations for the treatment of light-induced skin damages, the co-antioxidants should guarantee its photostability beside preserving the catechin chemical stability. The results reported in this study indicated that vitamin E and BHT, two of the most commonly used antioxidant additives for EGCG chemical stabilization, do not protect the catechin from light-induced decomposition. On the other hand, an equimolar concentration of α-lipoic acid was found to inhibit almost completely the photodegradation of EGCG and to preserve its functional activity (antioxidant capacity) under solar irradiation. Therefore, the correct selection of the co-antioxidant stabilizer is of paramount importance to ensure the therapeutic activity of EGCG under solar irradiation. In addition, α-lipoic acid as photostabilizer of EGCG is much more efficient and it is effective at a lower concentration than the previously reported sunscreen agents [18]. *In vivo* studies should be performed in order to assess the relevance of the observed stabilization effect provided by α-lipoic acid *in vitro*, to the actual conditions of use of topical EGCG preparations.

The results obtained in the present study, also suggest that EGCG is particularly suitable as antioxidant agent in topical preparations for protection of the skin from the adverse effects induced by solar UV radiation. In fact, at variance with other commonly used reducing agents, EGCG retains some of its antioxidant properties under sunlight, despite undergoing marked photodecomposition. 
